# Prevalence and Factors Associated with Teenage Pregnancy, Northeast Ethiopia, 2017: A Cross-Sectional Study

**DOI:** 10.1155/2018/1714527

**Published:** 2018-11-01

**Authors:** Yohannes Ayanaw Habitu, Anteneh Yalew, Telake Azale Bisetegn

**Affiliations:** ^1^Department of Reproductive Health, College of Medicine and Health Sciences, University of Gondar, Gondar, Ethiopia; ^2^Wogedi District Health Office, Wogedi, South Wollo Zone, Northeast Ethiopia, Ethiopia; ^3^Department of Health Communication and Behavioural Sciences, Institute of Public Health, College of Medicine and Health Sciences, University of Gondar, Gondar, Ethiopia

## Abstract

**Introduction:**

Though teen age pregnancy had poor maternal and perinatal health outcomes, its magnitude and determinants are not well understood. Therefore, the aim of this study was to assess the prevalence and associated factors of teenage pregnancy in Wogedi, northeast Ethiopia.

**Methods:**

A community-based cross-sectional study was conducted among 514 teenagers in Wogedi, northeast Ethiopia, from April to May 2017. Data were collected using a structured questionnaire, entered, and analyzed appropriately. Odds ratios with 95% confidence interval and P-values were computed using appropriate logistic regression models to determine the presence and strength of associations between the dependent and independent variables.

**Results:**

The prevalence of teenage pregnancy in Wogedi was 28.6% (95% CI: 24.9, 32.5). Age (AOR=2.10; 95% CI: 1.55, 2.88), rural residence (AOR=3.93; 95% CI: 1.20, 12.83), contraceptive nonuse (AOR=10.62; 95% CI: 5.28, 21.36), and parental marital status (divorce) (AOR=1.98; 95%CI: 1.13, 3.93) were found to have statistically significant associations with teenage pregnancy.

**Conclusions:**

There is high prevalence of teenage pregnancy in the area. Age, residence, contraceptive nonuse, and parental divorce were found to have a statistically significant association. Strengthening contraceptive use by giving special attention to rural dwellers and showing the consequences of divorce to the community are strongly recommended.

## 1. Introduction

Adolescent pregnancy is defined as a pregnancy in girls 10–19 years of age. It is estimated that about 16 million girls 15–19 years old give birth each year, contributing nearly 11% of all births worldwide [1]. Although adolescent fertility rates are falling globally, approximately 18 million girls under the age of 20 give birth each year [2]. Two million of these births are from girls under 15 years of age [2]. More than 90% of these births occur in low and middle-income countries [1–3]. Most teenage pregnancies and childbirths take place in west and central Africa, east and southern Africa, South Asia, Latin America, and the Caribbeans [2].

Different pieces of literature show that the prevalence of teenage pregnancy varies across regions of the world. In the Asia Pacific regions, it ranges up to 43% in Bangladesh [4] and from 11.1% [5] to 47.3% in Nepal [6]. In Jordan, the prevalence is 25% [7]. The prevalence of teenage pregnancy also varies in Africa; for instance, in Nigeria, it ranges from 6.2% in Niger Delta state [8] to 49% in Abia State [9]. In South Africa [10], East Africa (Kenya) [11], Assossa (Ethiopia) [12], and Sudan [13], it ranges from 2.3 to 19.2%, 31%, 20.4%, and 31%, respectively.

Currently about 17% of the adolescents between 15 and 19 years in Ethiopia are married [14] and the median age of women at first sexual intercourse is now 16.6 years [14]. There is a low contraceptive prevalence rate (CPR) (7.5%) among all female adolescents of 15-19 years; CPR is higher among currently married (31.9%) and sexually active unmarried adolescents (59%) of the same age [14]. In addition, 20.5% of female adolescents 15-19 years face unmet needs for family planning and 52.5% total demand for same [14].

Teenage pregnancy is very common in Ethiopia, and it is an important demographic factor making the country the second most populous in Africa, with a total population of around 102 million in 2016 [15]. According to the EDHS 2016 finding, the prevalence of teenage pregnancy is 13% [14]. It varies depending on residence, urban 5%, and rural 15% [14]. Moreover, disparities are seen across regions, with the highest 23% in Afar, 8% in Amhara, and the lowest 3% in Addis Ababa [14].

Literature showed different sociodemographic, cultural, and other individual factors were associated with teenage pregnancy. Approximately 90% of teenage pregnancies in the developing world are of girls who are married, owing to their high exposure to sex and pressure to conceive quickly after marriage [8, 16–18]. As a result, the majority (75%) of married teenage pregnancies are planned [1, 17]. Employment status [6, 9, 18, 19], poverty [2, 6, 14, 20–22], marital status [9, 12, 18, 23], type of occupation [12, 21], culture [9, 11], peer pressure [11, 20, 22, 24], early marriage [25], forced marriage [20], rape [26], and the need for a dowry [20] were factors associated with teenage pregnancy in recorded literature.

Studies have shown that teenage pregnancy has poor maternal and perinatal health outcomes [1, 2, 8, 27]. Complications during pregnancy and childbirth are the second cause of death for 15-19-year-old girls globally [1]. Every year, some 3 million girls aged 15 to 19 undergo unsafe abortions [1]. Babies born to adolescent mothers face a substantially high risk of dying than those born to women aged 20 to 24[1, 25]. School dropout [14, 20, 22, 25], poverty [2, 25], high rate of marriage [8], pregnancy-induced hypertension [27–30], and induced abortion [1, 20] are some of the consequences of adolescent pregnancy on the mother. Preterm delivery [19, 27, 29–31], low birth weight [5, 19, 27, 29–32], stillbirth [5, 27, 31], and high fetal and neonatal mortality [19, 27, 29, 30, 32] are some of the consequences of teenage pregnancy for the fetus.

The limited studies conducted in the world as well as in Ethiopia have tried to show the prevalence and factors associated with teenage pregnancy, but most of the studies were done using secondary data (health facility-based studies). Hence, due to the poor data recording system of our country, we may not get appropriate information from health facilities. Moreover, research results from health facilities lack representativeness to the general population. Therefore, this study was conducted to show the prevalence and determinants of teenage pregnancy in Wegedi, northeast Ethiopia, 2017, by using primary data.

## 2. Materials and Methods 

### 2.1. Study Design and Setting

This community-based cross-sectional study was conducted in Wogedi from April to May 2017. Wogedi, one of the districts in South Wollo zone, is 192 km to the west of Dessie. According to the national census report of 2007, the projected population of Wogedi for the year 2016 was 152,719 of whom 26.5% were adolescents 15-19 years of age [33]. Wogedi has 35 kebeles (lowest administrative units), two district health offices, five health centers, and thirty-two health posts [33].

### 2.2. Source and Study Population

All female adolescents 15-19 years of age in the selected kebeles of Wogedi were the source population of the study.

### 2.3. Inclusion Criteria

Female adolescents 15-19 years of age.

### 2.4. Sample Size and Sampling Procedure

The sample size was calculated using the single population proportion formula with the following assumptions. The proportion of teenage pregnancy among 15-19 years of age females in Ethiopia (20.4%) [34] was taken from the previous study with a 5% margin of error and 95% confidence interval. Lastly, by considering a 10% nonresponse rate, and a design effect of 2, the final sample size obtained was 542. A multistage sampling technique was employed in order to select study units. First, out of the 35 kebeles, six were selected randomly, to represent 20% of the district population. Then, to each selected kebele, we allocated the sample size by considering the proportional allocation technique. Finally, study units were randomly selected at the household level using the lottery method.

### 2.5. Study Variables

#### 2.5.1. Dependent Variable

Teenage pregnancy.

#### 2.5.2. Independent Variables

Sociodemographic variables, like age, sex, marital status, education, occupation, and income were considered. History of sexual and reproductive health, like age at first sexual intercourse, early marriage, contraceptive use, perception on teenage pregnancy, family income, family education, peer pressure, and casual sex assessed.

### 2.6. Operational Definition

*Teenage pregnancy:* pregnancy in adolescents 15-19 years who believe that they are pregnant and confirmed by a health care providers.

### 2.7. Data Collection Instruments and Procedures

Data were collected using a pretested, structured, interviewer-administered questionnaire which was first prepared in English and translated into Amharic (the local language) by a language expert. Then, the Amharic version was again translated back to English to check for consistency. The structured, interviewer-administered questionnaire was adapted from the WHO (Illustrative-questionnaire for interview survey with young people developed by John Cleland) standard tool which was developed to assess the sexual and reproductive health of adolescents and youth. Appropriate modifications were made to fit with the local set up. In addition we conducted a pretest and we have made some simple analysis to see if we can address the desired objectives or not, from the results of the pretest. Moreover, some language corrections and rearrangements on the order of questions were made to keep the logical flow of the questions, based on the comments from the pretest we got. Four data collectors, with Bachelor's degrees in nursing and data collection experience were recruited. One supervisor, with a master's of public health was employed. The supervisor and data collectors were females chosen in order to minimize participant discomfort. A one day training was given to data collectors and the supervisor to help them know the objective and the relevance of the study and the rights of the respondents either to participate or decline.

### 2.8. Data Management

Data manually checked by the investigators were entered and cleaned using EPI info data version 7 and exported to SPSS version 20 for further analysis. Descriptive statistics like frequencies, percentages, and means were computed. Bivariable analyses were carried out to examine the relationship between teenage pregnancy and each explanatory variable. All explanatory variables that were statistically significant at the Bivariable model at <0.2 p-value were included in the multivariate logistic regression model to see the real determinants of teenage pregnancy. Adjusted odds ratios with a 95% confidence interval were computed, and variables with a p-value <0.05 in the Multivariate Model were considered as statistically significant.

### 2.9. Ethical Considerations

Ethical approval was obtained from the Institutional Review Board (IRB) of the Institute of Public Health, College of Medicine and Health Sciences, the University of Gondar (IRB reference number: IPH/2471/2017). Participants were informed about the objective of the study and the fact that the confidentiality of their responses will be insured by excluding personal identifiers and carefully securing the questionnaire in such a way that it is accessed only by the investigators.

## 3. Results

### 3.1. Sociodemographic Characteristics of Respondents

A total of 514 female adolescents 15-19 years of age were included in the study with a response rate of 95%. The majority, 157 (30.5%), of the respondents were 19 years old, with the median Inter Quartile Range of 3 years. More than half, 270 (52.5%), of the respondents were Orthodox Christians. Three hundred seventeen, (61.7%), attended primary school, and more than half, 271 (52.7%), were married. Nearly half, 253 (49.2%), of the respondents were students, and 251 (48.8%) earned less than Birr 1500 per month (Table 1).

### 3.2. Sexual and Reproductive Health Characteristics of Participants

This study showed that 337 (65.6%) of the respondents had sexual intercourse and that 130 (38.6%) started it before 15 years of age. Out of those who had sex, 156 (46.3%), used contraceptives. The proportion of teenage pregnancy among respondents in Wogedi was 147 (28.6%) with a 95% CI (24.9, 32.5). The study also indicated that the majority of the pregnancies, 93 (63.3%), were unplanned, and 54 (36.7%) of the teenagers were unhappy about their pregnancy. Moreover, 36 (24.5%) were pregnant at the time of the survey (Table 2).

The major reason mentioned by 65% of the respondents for exposure to pregnancy was marriage, followed by 20% who were exposed for nonuse of contraceptives.

### 3.3. Factors Associated with Teenage Pregnancy

Age, religion, educational status, marital status, occupation, living arrangement, parent's age, parents' religion, parents' marital status, parents' monthly income, and contraceptive nonuse were variables with P-values of less than 0.2 in the Bivariable Logistic regression. All variables with less than 0.2 P-values were fitted in to the multivariate logistic regression model after adjusting for possible confounders. In the multivariate logistic regression model, age, residence, contraceptive nonuse, and parents' marital status were identified as statistically significant variables.

Increase in age is significantly associated with teenage pregnancy (AOR=2.10; CI: 1.55, 2.88). Teenagers from rural settings were more likely to be pregnant than students (AOR=3.93; 95% CI: 1.20, 12.83). This study also showed that contraceptive nonuse was found to be significantly associated with teenage pregnancy. Respondents who did not use contraceptives were ten times (AOR=10.62; 95%CI: 5.28, 21.36) more likely to be pregnant than their counterparts.

Teenagers from divorced parents were nearly two times more exposed to teenage pregnancy as compared to those who were from married parents (AOR=1.98; 95%CI: 1.13, 3.93) (Table 3).

## 4. Discussion

The prevalence of teenage pregnancy extremely varies in the world. Some of the reasons for these differences could be variations in sociodemographic, cultural, sexual, and reproductive health characteristics of adolescents. This study explored the prevalence of teenage pregnancy and its associated factors in Wogedi district, northeast Ethiopia. The study showed that the prevalence of teenage pregnancy was 28.6% (95% CI: 24.9, 32.5). Factors associated with it were increase in age, farming occupation, contraceptive nonuse, and parents' marital status (divorce).

This finding is similar with those of studies conducted in Sudan, 31% [13], Kenya, 31% [11], Jordan, 25% [7], and Turkey, 29% [35]. This similarity could be due to the presence of some related sociodemographic, cultural, and individual adolescent characteristics in the current and those studies. For instance, the proportion of married adolescents in the Kenya study was similar to that of the current study. Moreover, there was a similarity in the culture of early marriage in the current and the other studies.

This finding is higher than the 13.0% national report on teenage pregnancy [14]. The possible reason may be that the EDHS study includes all settlements, like urban and rural areas, whereas the current study was conducted in one of the rural districts in Amhara Region, where there is a high prevalence of early marriage. In the region, the median age at first marriage is 16.2 years, and the median age at first sex 15.8 years, the lowest in the country [14]. All these factors may contribute to the high prevalence of teenage pregnancy in the current study as compared to the national one.

This finding is higher than those of studies conducted in Niger Delta state of Nigeria 6.2% [8], and Assosa 20.4% [12]. The variation could be due to the presence of some sociodemographic, cultural, sexual, and reproductive characteristics of participants in the other and the current study. For instance, the proportion of marriage in the Niger Delta state was 27.7% [8], lower than that of the current study 39.9%. Shreds of evidence showed that as the proportion of marriage increased, the probability of exposure to pregnancy also increased [36]. In addition, the proportion of participants who had secondary or more education was higher in the Niger Delta state study (46.0%) [8] compared to the current study (20.6%). Evidence showed that as the educational level of girls increased, the chance of exposure to pregnancy decreased [14]. Compared to marriage in the current study (39.9%), the proportion of marriage was lower in the Assosa study 20.7% [12]. In addition, the proportion of participants who could not read and write was lower in the Assosa study (3.4%) [12] compared to that of the current study (17.7%). Moreover, the proportion of contraceptive use was higher (69.5%) in the Assosa study [12] compared to the present study (46.3%). Different scientific researchers showed contraceptive use decreased the prevalence of teenage pregnancy [36].

This finding is lower than those of studies conducted in Abia State of Nigeria (49.0%) [9] and the Cape Coast Municipality in Nepal (47.3%) [6]. This difference could be due to some social, cultural, and individual characteristics of participants and the time gap among the studies. For instance, in the Abia study, the proportion of participants who had no formal education (28.5%) [9] was more than that of the current study which represented 17.7%. Moreover, the proportion of study participants who were unemployed (75.8%) was higher in Abia State study [9] compared to that of the present study (49.2%). The possible explanation for these may be unemployed adolescents may have poorer access to contraceptives; hence, the chance of getting pregnancy increased. In the study conducted in Cape Coast Municipality, Nepal, the proportion of participants who had no formal education (30.2%) [6], was higher compared to that of the current study (17.7%). Furthermore, the proportion of participants who had secondary education and above (2.1%) [6] was less in the Nepal study compared to that of the present study (20.6%). Besides, the proportion of contraceptive use in the Nepal study 11.1% [6] was less than that of the present study (53.7%).

In this study, as respondents' age increased by one year, the odds of being pregnant increased by 2.1%. This finding is in line with that of national study in Ethiopia [14], Kenya [11], and Abia State [9]. As age increases, teenagers will have more exposure to sex and their chance of being married will also increase to procreate children.

Teenagers from rural setups were four times more likely to have pregnancy compared to student respondents. This finding is consistent with that of a study conducted in South Asia [37]. This might be so because teenagers from the rural areas are less educated and have limited access to contraceptives. Besides, rural communities are pronatalists compared to educated people [14].

Contraceptive nonusers were nearly eleven times more likely to be pregnant compared to those who used contraceptives. Other studies are in line with this finding in that the prevalence of teenage pregnancy increased among contraceptive nonusers [18, 20, 36]. It has been evidenced that as the proportion of contraceptive nonusers increased the proportion of pregnancy increased [36].

Teenagers from divorced parents were nearly two times more exposed to teenage pregnancy compared to adolescents from married parents (AOR=1.98; 95%CI: 1.13, 3.93). This is due to the existence of low parental control and communication about sexual and reproductive issues among divorced parents compared to married ones. These lead to increased early sexual debut and risky sexual behaviors among adolescents from divorced parents, and all these expose them to teenage pregnancy [38–40]. Since the study design employed is a cross-sectional, causality may not be inferred. Social desirability bias is one of the potential biases, which may affect the results of this study. To minimize the effect of social desirability bias we recruited data collectors who have no close contact with the respondents.

## 5. Conclusions

This study showed that there is a high prevalence of teenage pregnancy in the area. Increased age, rural residence, contraceptive nonuse, and parental marital status (divorce) were found to have a statistically significant association with teenage pregnancy. Strengthening contraceptive service promotion and provision for teenagers by giving special attention to rural ones and showing the consequences of divorce in the community are strongly recommended.

## Figures and Tables

**Table 1:** Sociodemographic characteristics of teenagers in Wogedi, northeast Ethiopia, 2017.

**Variables **	**Frequency (N=514)**	**Percent**
**Age in years**		
15	86	16.7
16	83	16.2
17	76	14.8
18	112	21.8
19	157	30.5
**Religion **		
Muslim	244	47.5
Orthodox	270	52.5
**Marital status**		
Married	205	39.9
Divorced	63	12.3
Widowed	3	0.6
Single	243	47.2
**Educational status**		
College	5	1.0
Secondary (9-12)	101	19.6
Primary (1-8)	317	61.7
Unable to read and write	91	17.7
**Occupation **		
Farmer	173	33.7
Housewife	39	7.6
Merchant	18	3.5
Daily laborer	31	6.0
Student	253	49.2
**Live with**		
Alone	16	3.1
Husband	164	31.9
Parent	334	65.0
**Expected household income**		
<1500	251	48.8
1501-7500	244	47.5
>7500	19	3.7

**Table 2:** Sexual and reproductive health characteristics of teenagers in Wogedi, northeast Ethiopia, 2017.

**Variable **	**Frequency**	**Percent**
**Ever had sex (N=514)**		
No	177	34.4
Yes	337	65.6
**Age at first sex (N=337)**		
13-15	130	38.6
16-18	205	60.8
>18	2	0.6
**Contraceptive use (N=337)**		
No	181	53.7
Yes	156	46.3
**Reasons for contraceptive non-use (N=181)**		
Do not have accesses	17	9.4
Do not have knowledge	68	37.6
Family influence	81	44.8
Due to divorce	8	4.4
Wants to be pregnant	7	3.8
**Ever had pregnancy (N=514)**		
No	367	71.4
Yes	147	28.6
**Currently pregnant (N=147)**		
No	111	75.5
Yes	36	24.5
**Conditions of pregnancy (N=147)**		
Planned	54	36.7
Unplanned	93	63.3
**Feeling about the pregnancy (N=147)**		
Happy	58	39.5
Unhappy	64	43.5
Nothing	25	17.0
**Outcomes of pregnancy (N=111)**		
Delivered (Live Birth)	97	87.4
Aborted	14	12.6

**Table 3:** Bivariable and multivariable logistic regression analysis showing factors associated with teenage pregnancy in Wogedi, northeast Ethiopia, 2017.

**Variable **	**Teenage pregnancy**		**Crude OR (95**%** CI)**	**Adjusted OR (95**%** CI)**
	**Yes **	**No **		
**Age** (Mean =17.33 years with SD=1.47)	147	367	2.60 (2.12,3.20)	**2.10 (1.55,2.88)** **∗** **∗**
**Marital status**				
Married	111	94	30.7 (14.9, 41.0)	2.16 (0.59, 4.80)
Divorced	24	39	16 .0 (6.9, 36.1)	1.38 (0.37, 5.00)
Widowed	3	0	-	-
Single	9	234	**1**	**1**
**Educational level**				
College	2	3	0.5 (0.08, 3.13)	3.00 (0.28, 6.1)
Secondary	4	97	0.03 (0.01, 0.09)	0.87 (0.42, 1.78)
Primary	89	228	0.29 (0.18, 0.47)	0.41 (0.10, 1.67)
Unable to read and write	52	39	**1**	**1**
**Occupation **				
Farmer	91	82	30.00 (14.5,62.3)	**3.93 (1.20,12.83)** **∗**
House wife	28	11	69.01 (26.3,80.0)	4.23 (0.94,19.01)
Merchant	12	6	54.22 (16.5,77.0)	4.26 (0.75, 6.13)
Daily laborer	7	24	7.91 (2.73, 23.1)	1.42 (0.31, 6.57)
Student	9	244	**1**	**1**
**Live with**				
Alone	11	5	16.62 (5.43, 20.04)	3.24 (0.55, 8.83)
Husband	97	67	10.91 (6.93,17.01)	2.26 (0.81, 6.28)
Parents	39	295	**1**	**1**
**Monthly income**				
<1500	81	170	0.65 (0.25, 1.69)	2.59 (0.69, 9.61)
1501-7500	58	186	0.42 (0.16, 1.12)	1.33 (0.36, 4.96)
>7500	8	11	**1**	**1**
**Parent age**				
30-49	95	265	0.7 (0.46, 1.04)	0.96 (0.49, 1.86)
>49	52	102	**1**	**1**
**Parents' marital status**				
Divorced	7	29	2.2 (0.72, 6.90)	**1.98 (1.13, 3.93)** **∗**
Widowed	14	36	2.42 (0.91, 6.32)	1.54 (0.12, 18.95)
Married	126	302	**1**	1
**Contraceptive use**				
No	117	64	7.6 (4.65, 12.67)	**10.62 (5.28, 21.36)** **∗** **∗**
Yes	30	126	**1**	**1**

*∗*Significant at a p-value less than 0.05 and *∗∗*significant at a p-value less than 0.001; SD: Standard Deviation.

## Data Availability

The data used to support the findings of this study are included with in the article.

## Abbreviations

AOR: Adjusted odds ratio
CI: Confidence interval
COR: Crude Odds Ratio
CPR: Contraceptive prevalence rate
EDHS: Ethiopian Demographic Health Survey
SPSS: Statistical Package for Social Science.
